# A Model for Estimating the Burden of Disease of Transfusion-Transmitted Infection

**DOI:** 10.3389/ijph.2024.1607165

**Published:** 2024-08-06

**Authors:** William Riley, Kailey Love, Mary Saxon, Aaron Tobian, Evan M. Bloch, Ronnie Kasirye, Irene Lubega, Ezra Musisi, Aggrey Dhabangi, Dorothy Kyeyune, Jeffrey McCullough

**Affiliations:** ^1^ College of Health Solutions, Arizona State University Downtown Phoenix Campus, Phoenix, AZ, United States; ^2^ Sandra Day O’Connor College of Law, Arizona State University, Tempe, AZ, United States; ^3^ School of Medicine, Johns Hopkins University, Baltimore, MD, United States; ^4^ Makerere University - Johns Hopkins University Research Collaboration, Kampala, Uganda; ^5^ Uganda Blood Transfusion Services, Kampala, Uganda

**Keywords:** transfusion-transmitted infections, blood transfusion, burden of disease, Uganda, disability adjusted life-years

## Abstract

**Objectives:**

Blood transfusion is an important mode of infectious disease transmission in low- and middle-income countries (LMICs). This study describes a model to determine the prevalence of transfusion-transmitted infections (TTIs) and the associated burden of disease.

**Methods:**

A five-step model was developed to determine the TTI-related burden of disease measured by disability-adjusted life years (DALYs). Uganda was selected as the study country.

**Results:**

Approximately 298,266 units of blood were transfused in Uganda in 2019, yielding an estimated TTI incidence of 6,858 new TTIs (2.3% of transfused units) and prevalence of 19,141 TTIs (6.4% of transfused units). The total burden of disease is 2,903 DALYs, consisting of approximately 2,590 years of life lost (YLLs), and 313 years lived with disability (YLDs).

**Conclusion:**

The incidence and prevalence of TTIs and the associated burden of disease can be calculated on a local and national level. The model can be applied by health ministries to estimate the impact of TTIs in order to develop blood safety strategies to reduce the burden of disease.

## Introduction

Infectious diseases take a large toll in developing nations, representing 68% of all deaths in Africa [1]. Transfusion-transmitted infections (TTIs) are an important subset of infectious diseases and contribute to the overall burden of disease, adversely impacting transfusion recipients in low and middle-income countries (LMICs). While there has been progress in decreasing TTIs in high-income countries, blood safety in low-income countries remains a major challenge [2, 3]. The lack of an adequate and safe blood supply is a major impediment to health in many LMICs, with a considerable risk of TTIs in sub-Saharan Africa [4]. This is primarily due to a high prevalence of infectious diseases among the general population, the use of paid or replacement donors as well as inconsistent and incomplete laboratory screening of the blood supply [1–6].

The World Health Organization (WHO) recommends testing all donated blood for the presence of the major TTIs which includes human immunodeficiency virus (HIV), malaria, hepatitis E virus (HEV), hepatitis B virus (HBV), hepatitis C virus (HCV), and syphilis to improve blood safety and reduce the burden of disease [7]. Malaria remains a major TTI and is screened by travel history triggered deferral and not laboratory testing [8]. Mitigation strategies for transfusion transmitted malaria differ between endemic and non-endemic countries [9]. In non-endemic countries, risk-based deferral is typical. In endemic areas, there is limited screening (e.g., Eliminating febrile donors, using microscopy or antigen testing). This is known to be suboptimal. Almost all high-income countries comply with the WHO guidelines to ensure blood safety, and there has been progress in many low-income countries [10, 11]. However, blood safety remains a major challenge in LMICs where it is estimated that 40% of donated blood may not undergo testing for all the major TTIs [12, 13].

The prevalence of infectious diseases has been studied extensively; however, there is very little information regarding the impact of TTIs on the burden of illnesses and population health. This study develops a model to estimate the burden of disease for TTIs.

## Methods

The research approach for this study involved five procedures: 1) identify infectious pathogens transmissible through blood transfusions, 2) select an appropriate database for the relevant variables for each TTI, 3) determine the prevalence and incidence of each TTI, 4) estimate the number of infectious units of blood transfused, and 5) determine the burden of disease from each TTI using Years of Life Lost (YLL) to premature death, the number of Years Lived with Disability (YLD), and the Disability-Adjusted Life Years (DALY). This study is part of a larger randomized control trial, Mirasol Evaluation of Reduction in Infections Trial (MERIT), to evaluate the reduction of TTIs in Uganda [14]. The larger MERIT study was reviewed and approved by two Institutional Review Boards (IRB). The present study was deemed exempt by the IRB.

### Infectious Diseases Transmissible Through Blood Transfusions

Uganda data were used to develop the model. The model was first developed by delineating infections that are transmissible through blood transfusions as well as their population incidence and prevalence. In the larger study, five transmissible pathogens were included but two TTIs were not - trypanosoma cruzi due to a lack of donor prevalence estimates and syphilis because TTI risk is difficult to estimate due to the relationship between disease stages and pathogen transmission. For blood donors, HIV, HBV, and HCV were screened by testing, malaria was screened by donor history, while Hepatitis E (HEV) was not screened because HEV is not considered a risk of blood transfusion and therefore screening is not done. While some units of blood are contaminated with bacteria [15], this infection source was not included in the model because the YLD, YLL, and DALY are not available from the data sources for this study. Blood donor screening and seroprevalence data were obtained in the larger study from the Uganda Blood Transfusion Service in Kampala, Uganda between April – June 2017 [16]. The screening methods used by Uganda National Blood Transfusion Service (UNBTS) are the Abbott Architect, an immunoassay analyzer, for HIV, HBV, and HCV [17]. The number of infectious units missed by the screening tests were calculated from the number of positive tests and the test sensitivity of each method.

### Database for Transfusion-Transmitted Infection Impact on Health Burden

The Institute for Health Metrics and Evaluation (IHME) Global Burden of Disease (GBD) dataset was selected as the primary data source for estimating incidence, population prevalence, and burden of disease metrics. The IHME GBD dataset is the largest and most comprehensive effort to quantify health loss, leveraging 328,938 data sources, and is the only health impact datasource available for the diseases in this project [18, 19]. Three IHME GBD variables were used in this model: Years of Life Lost (YLL) due to premature mortality; Years Lived with a Disability (YLD); and Disability-Adjusted Life Years (DALYs) [20] 2019 Uganda data for YLLs, YLDs, and DALYs were used in this model.

The burden of disease refers to the sum of mortality and morbidity from illness and is often measured as DALYs [20]. The DALYs describe death and loss of health due to diseases and is a method used by the WHO to estimate the impact of death and disability of a specific disease [21]. The burden of disease was estimated by adding together the number of years of life lost (YLL) to premature death and the number of years of life a person lives with a disability caused by the disease (YLD) and the overall impact was measured by the disability-adjusted life years (DALY).

YLL is a measure of premature mortality calculated by deducting the age of death from the life expectancy for that individual. For each disease, the age at death and life expectancy were obtained from the IHME GBD database using 2019 data [22]. This study estimated the YLLs associated with each TTI by multiplying the age-adjusted YLL with the disease prevalence.

YLD estimates the number of years that an individual lives with a functional impairment caused by a disease. The YLD is calculated by multiplying the disease prevalence by the loss of health associated with the disability (disability weight) [23]. The disability weight reflects the severity of the disease and is measured between 0 (perfect health) to 1 (equivalent to death) and was adapted from the estimate developed by the IHME. This study estimated the YLDs associated with each TTI by multiplying the age-adjusted YLD per person for each disease by the prevalence of disease-related TTIs.

DALYs represent the loss of the equivalent of 1 year of full health either by death or disability [21]. The DALY is a foremost measure to estimate the number of years that an individual lives with a functional impairment caused by a disease. This study estimated the YLDs associated with TTIs by multiplying the age-adjusted YLDs per person for each disease by the prevalence of overall disease-related TTIs, estimated for the burden of disease. This study also estimated the DALY associated with the TTI for each disease by summing the TTI-related YLLs with the TTI-related YLDs.

### The Prevalence and Incidence of TTIs

Data regarding the positive test rates in donors and the number of units distributed to facilities were obtained from the Uganda Blood Transfusion Service (UBTS) [14]. An additional data source includes the manufacturer’s instructions for testing methods to provide test sensitivity and specificity [17]. This study estimated the incidence and prevalence of TTIs in 2019 using the following steps: A) determine the number of positive screening tests for infectious agents in the blood donor pool; B) identify the sensitivity of the screening test; C) calculate the number of infectious units missed by the screening test; D) identify the infectivity of the infectious agent; E) estimate the number of infectious units of blood transfused; and F) calculate the incidence and prevalence of TTIs based on number of units of blood transfused.

As recommended in other studies, this study used the test sensitivity provided by the manufacturer. It is possible that in practice these sensitivity levels may be lower. The likelihood of developing disease from infection with these agents will depend on many factors such as limited quality assurance/quality control programs.

The total number of infectious units transfused (Step F) were calculated as shown in Formula One. The incidence and prevalence of the five TTIs was obtained from the IHMD GBD database [19]. The number of units transfused per person is unknown but—for the purposes of this modeling study—it was assumed that no patient received more than one infected unit. Based on the known infectivity, the number of patients with actual disease was then calculated (Table 2). This provides the incidence of new infections.

Finally, the incidence and prevalence of TTI’s was age adjusted. The final calculation estimated the incidence and prevalence of TTIs adjusted for age. The age distribution of transfusion recipients was determined from a blood utilization study in Uganda [24].

Formula One: Total Number of Infectious Units Transfused

$$\text{Expected Number of Infected Transfused Units}=\left(\left[\text{Infectious Units}/1,000\text{ Units of Blood}\right]x\text{ Units Transfused Annually}\right)$$

Formula two demonstrates the estimation of the TTI incidence by age, achieved by multiplying the expected infectious units per 1,000 transfusions for each disease by the number of transfused units in the corresponding age category.

Formula Two: Incidence of TTIs

Incidence of TTIs by Age of Patient=Number of Transfusions×Number of Expected Infections by Disease Type Adjusted for Age

Formula three outlines the calculation for the age adjusted TTI prevalence. This was done by multiplying the TTI incidence by the overall disease incidence/prevalence ratio for the specific infection within the age-group. This approach was repeated for a total of five infectious diseases.

Formula Three: Age-Adjusted Prevalence of TTI

$$\text{Age}-\text{Adjusted Prevalence of TTI}=\text{TTI Incidence}\times \left(\text{Overall Disease Incidence}/\text{Overall Disease Prevalence}\right)$$

## Results

Table 1 describes the population incidence and prevalence of the five TTIs. The incidence of these five diseases in the Ugandan population is 11.5 million persons (28% of total population) and the prevalence of the five diseases is 15.6 million persons (38% of the total population). The infectious disease prevalence ranges from a high of 14.1 (34.4%) million for malaria to a low of 5,343 for HCV (0.01%).

**TABLE 1:** Population incidence and prevalence of five infectious diseases in Uganda that can be acquired through transfusions (Uganda, 2019).

	Incidence^a^		Prevalence^a^	
Disease	Population incidence of infection^b^	Percentage of total population (%)	Population prevalence of infection	Percentage of total population (%)
HIV	79,385	0.2	1,366,481	3.3
Malaria	10,706,227	26.0	14,144,213	34.4
HEV	164,637	0.4	12,619	0.03
HBV	539,920	1.3	62,298	0.2
HCV	46,308	0.1	5,343	0.01
Total	11,536,477	28.06	15,590,955	37.92

^a^ Prevalence and Incidence 2019 Data from the Institute for Health Metrics and Evaluation Global Burden of Disease [19].

^b^ Total Uganda Population Estimate 2019: 41.1 million19.

Table 2 shows the prevalence of infections in blood donors for the five diseases and was adjusted for screening sensitivity as well as infectivity to calculate the expected number of TTIs per thousand transfused individuals. Using the donor test positive rate and the screening sensitivity, there is an estimated total of 57 infectious units per 1,000 units donated, ranging from 0.2 (HIV) to 50 (malaria) potential infections per 1,000 units. Adjusting for infectivity ranging from 20% (malaria) to 100% (HIV), there would be a total of 18 infected patients per 1,000 units of blood transfused ranging from 0.3 for HIV to 10 for malaria.

**TABLE 2:** Transfusion-transmitted infections in Uganda among blood donors^a^ (Uganda, 2019).

Disease	Seroprevalence in donor population (%)	Screening method sensitivity (%)	Infectious units/1,000 units of blood	Infectivity (%)	Expected number of infections/1,000 transfused individuals +
HIV^b^	0.71	98	0.2	100	0.3
Malaria^c^	5.0	No Screening	50	20	10
HEV^b^	0.6	No Screening	6	75	6.8
HBV^b^	2.14	98	0.4	75	0.5
HCV^b^	1.76	98	0.4	74	0.4
Totals	-	-	57	-	18

^a^ Table modified from (Kasirye et. al) Mirasol Evaluation of Reductions in Infections Trial Study [22].

^b^ Seroprevalence data from Uganda Blood Transfusion Service in Kampala, Uganda between April – June 201716.

^c^ Malaria donor prevalence of 5% is a conservative estimate from data in Uganda [24].

Table 3 shows calculations of the age-adjusted incidence and prevalence of TTIs based on 298,266 transfused units. The total Incidence of TTIs is 6,858. The highest incidence of TTIs is malaria with 4,473 infections (65% of total TTI incidence). Malaria also has the highest age group incidence at 15–49 years with 2,484 TTIs (36% of the total). The total prevalence of TTIs is 19,141. Malaria has the highest prevalence at 16,991 (89% of total prevalence) and the highest TTI age group of ≥70 years with 7,063 TTIs (37% of total).

**TABLE 3:** Incidence and prevalence of transfusion-transmitted infections by age (Uganda, 2019).

		0–5 Years	6–14 Years	15–49 Years	50–69 Years	70+ years	Total
	Transfusions	41,174	37,975	165,624	34,466	18,987	298,266
HIV	Incidence of TTIs	(41,174) × (0.3/1,000) = 12	(37,975) × (0.3/1,000) = 11	(165,624) × (0.3/1,000) = 50	(34,466) × (0.3/1,000) = 10	(18,987) × (0.3/1,000) = 6	89
	Prevalence of TTIs	12/(5,773/17,251) = 37	11/(14,693/79,947) = 62	50/(54,982/1,023,920) = 925	10/(3,554/1226,646) = 659	6/(383/18,717) = 279	1,962
Malaria	Incidence of TTIs	(41,174) × (15/1,000) = 618	(37,975) × (15/1,000) = 570	(165,624) × (15/1,000) = 2,484	(34,466) x (15/1,000) = 517	(18,987) × (15/1,000) = 285	4,473
	Prevalence of TTIs	618/(4,645,596/2,736,583) = 364	570/(3,284,036/4,953,989) = 859	2,484/2,676,733/5,687,901) = 5,279	517/(94,138/623,796) = 3,426	285/(5,724/141,945) = 7,063	16,991
HEV	Incidence of TTIs	(41,174) x (6.8/1,000) = 280	(37,975) x (6.8/1,000) = 258	(165,624) x (6.8/1,000) = 1,126	(34,466) x (6.8/1,000) = 234	(18,987) x (6.8/1,000) = 129	2,028
	Prevalence of TTIs	280/(45,024/3,418) = 21	258/(77,654/5,973) = 20	1,126/(39,207/3,016) = 87	234/(2014/155) = 18	129/(738/57) = 10	156
HBV	Incidence of TTIs	(41,174) x (0.5/1,000) = 21	(37,975) x (0.5/1,000) = 19	(165,624) x (0.5/1,000) = 83	(34,466) x (0.5/1,000) = 17	(18,987) x (0.5/1,000) = 9	149
	Prevalence of TTIs	21/(24,081/2,779) = 2	19/(47,731/5,507) = 2	83/(404,450/46,667) = 10	17/(53,658/6,191) = 2	9/(10,000/1,154) = 1	17
HCV	Incidence of TTIs	(41,174) × (0.4/1,000) = 16	(37,975) × (0.4/1,000) = 15	(165,624) × (0.4/1,000) = 66	(34,466) × (0.4/1,000) = 14	(18,987) × (0.4/1,000) = 8	119
	Prevalence of TTIs	16/(16,657/1,922) = 2	15/(10,354/1,195) = 2	66/(14,049/1,621) = 8	14/(4,095/472) = 2	8/(1,154.133) = 1	15
Grand Total Incidence of TTIs		947	873	3,809	792	437	6,858
Grand Total Prevalence of TTIs		426	945	6,309	4,107	7,354	19,141

The age at which a patient becomes infected will impact the YLL YLD and DALY. Table 4 estimates the years of life lost (YLL), years living with disability (YLD), and the disability-adjusted life years (DALY) for the 5 infectious diseases. For YLL, there are a total of 2,590 TTI-associated years of life lost, categorized by five diseases. HIV is the largest YLL with 1,508, (58%) of total TTI YLL. For YLD there are a total of 313 TTI-associated years living with disabilities, categorized by five diseases. HIV is also associated with the largest YLD with 191.34 (61%). For DALY there are a total of 2,903 disability-adjusted life years, caused by the five transmissible infections. The overall burden of disease related to TTIs is represented by 2,590 YLLs, 313 YLDs, and 2,903 DALYs.

**TABLE 4:** Transfusion-transmitted infections estimates for years of life lost, years lived with disability, and disability-adjusted life years for each disease (Uganda, 2019).

	HIV		Malaria		HEV		HBV		HCV		Totals		
	All causes	TTI	All causes	TTI	All causes	TTI	All causes	TTI	All causes	TTI	All causes	TTI	TTI% of total (%)
YLL	1,145,151	1,508	1,778,837	1,060	341	14.9	5,483	2	1,233	4.8	2,931,043	2,590	0.09
YLD	135,320	191.34	129,806	116.90	197	3.86	917	0.24	75	0.19	266,314	313	0.12
DALY	1,280,470	1,699	1,908,643	1,177	1,908,643	19	6,428	2.1	1,308	5.0	5,105,492	2,903	0.06

## Discussion

Blood transfusion is a lifesaving intervention for diverse conditions [25, 26]. However, it is not without risks, one of which is transmission of infection [27]. This study estimated the number of individuals who are infected through blood transfusions and the subsequent burden of disease in Uganda. The findings indicated that based on 298,266 transfused units in Uganda annually, there were approximately 6,858 (2.3%) TTIs. Malaria accounts for the majority (65%) of TTIs with an estimated 4,473 infections each year acquired from transfusion. This study did not attempt to determine the portion of patients who might already have been infected with malaria. It seems likely that at least some would have been infected previously with these agents; that is especially likely with malaria. Therefore, the apparent infections calculated here may overrepresent the total number of new infections. The model could account for these by applying prevalence data from the general population of these patients and determining the number who would be susceptible to new infections from transfusion.

Based on these results we estimated the TTI impact on the burden of disease and quality of life. Specifically, the estimated DALYs represent a loss of 2,903 years of full health in Uganda with 2,590 years of premature mortality (YLL) and 313 years of life lost due to disability (YLD). This disease burden may be compounded by the afflictions which necessitated the transfusions in the first place, and the loss of full health may be greater than estimated. This information is unknown and would be challenging to obtain given the wide range of underlying conditions that require transfusion and the potential added burden of the TTI. The findings suggest that TTIs have a substantial impact on the quality of life for Uganda’s population of 41 million persons.

Ensuring blood safety for recipients is an essential aspect of maintaining a stable blood supply [6]. TTI prevalence rates are lowest in high-income countries and highest in LMICs where as many as 25 percent of donations might not be screened [3]. Indeed, only 6 of 46 African countries reported 100% screening coverage for the major TTIs [12, 13]. In low-resource countries, the high prevalence of infectious diseases in the donor population adds an additional level of risk for ensuring blood safety.

This study used a narrow definition of TTI burden of disease based on the sum of mortality and morbidity. A broader definition of the burden of disease includes three major components: 1) the health impact caused by disabilities, 2) the loss of productivity, and 3) the cost of care and treatment [28]. All three components impede economic development. To fully understand the effect of death and loss of health due to diseases, it is important to consider both the burden of disease as well as its economic impact. While we estimate the burden of TTIs in this study, the model can also be used as a basis for an economic cost-benefit analysis of blood safety strategies. Future research should leverage this model to estimate the economic burden of TTIs.

The disability-adjusted life years (DALYs) reflect the disease burden associated with TTIs and highlight the importance of increased blood safety protocols in lower and middle-income countries. The DALYs is a relevant indicator for the burden TTIs impose on individuals and healthcare systems. By quantifying the impact of both premature mortality and disability caused by TTIs, the DALYs measure offers valuable insight into the magnitude of health challenges posed by these inflections.

### Limitations

There are several methodology limitations when estimating the burden of disease related to TTIs. First, the factors adopted from the IHME GBD were developed to estimate the burden of disease, rather than to estimate TTIs. Based on a careful review of IHME metrics and methodologies, we conclude that this approach can be adopted for use in this model. However, general donor TTI prevalence is lower than the general population. Second, the total population prevalence of HIV is underreported in our study (3.3%) compared to the World Bank database (5.5%) [29]. However, to maintain a consistent approach across the disease categories we used the IHME GBD database for all disease estimates. Third, syphilis was not included as a TTI due to infectivity being different at distinct stages of the disease. Fourth, the number of units of blood per transfusion and the number of transfusions per patient can increase or decrease the calculations for TTIs. We adjusted for the number of units per transfusion and the number of transfusions per person. Fifth, the use of secondary databases as a basis of our estimates introduces the parameters of those datasets. We used the age categories defined by the IHME. These age categories may not accurately reflect subsequent projections due to unknown distributions within the categories.

### Conclusion

The incidence and prevalence of infectious diseases such as HIV, Malaria, HBV and HCV are well established even in most LMICs, with a growing understanding of the impact of TTIs [30]. This study describes a model to estimate the extent of TTIs and their impact on the burden of disease in Uganda. The findings indicate that approximately 38% of the Ugandan population have a disease that is transmissible through transfusions and the incidence of infection through blood transfusion is 23 per 1,000 transfused individuals (6,858 TTIs incidence with 298,266 transfused units). This estimate that 2.3% of all patients who would have received transfusions contract a TTI is a substantial concern and leaves considerable room for improvement.

The model developed in this study offers a framework to evaluate the impact of transfusion transmissible diseases that can be used in high and low-resource countries. The risk of TTI is an ongoing challenge to blood safety; the effectiveness of TTI screening requires well-designed and implemented quality assurance systems. Screening for infectious blood donors primarily focuses on HIV, HBV, HCV, and syphilis [6]. However, the high rate of TTI suggests there is a considerable need to identify and implement methods to improve blood safety and reduce the burden of disease. Studies have explored the effectiveness of decreasing TTIs using pathogen reduction technology (PRT) [30–32]; and a large clinical trial is currently underway to establish its effectiveness in Uganda [22].

Understanding the impact of TTIs is important to ensure a safe blood supply and mitigate its contribution to ongoing transmission. This study introduced an approach to understanding the burden of disease associated with TTIs and can help prioritize efforts to improve blood safety. Future cost-benefit studies regarding the economic impact of transfusion-transmitted infections on developing nations can be used to evaluate various mitigation strategies.
